# Cognitive and visual task effects on gaze behaviour and gait of younger and older adults

**DOI:** 10.1007/s00221-023-06627-4

**Published:** 2023-05-06

**Authors:** Gregory S. Walsh, James Snowball

**Affiliations:** Department of Sport, Health Sciences and Social Work, Oxford Brookes University, Oxford, OX3 0BP UK

**Keywords:** Walking, Biomechanics, Inertial measurement unit, Eye tracking, Aging, Dual task

## Abstract

Cognitive dual tasks alter gait of younger and older adults and recent research has demonstrated that they also influence gaze behaviour and standing postural control. These findings suggest that age-related changes in cognitive and gaze function might increase fall risk in older adults. The purpose of this study was to determine the effect cognitive and visual dual tasks on the gait and gaze behaviour of younger and older adults. Ten older and ten younger adults walked for 3 min on a treadmill at preferred walking speed under three conditions, single task, cognitive and visual dual task conditions. Gait dynamics were measured using accelerometry and gaze behaviour was measured using wearable eye-trackers. Stride time variability and centre of mass (COM) motion complexity increased in dual-task conditions in older adults but had no difference for younger adults. Dual tasks had limited effect on gaze behaviour; however, visual input duration was greater, and visual input frequency and saccade frequency were lower in older than younger adults. The gaze adaptations in older adults may be the result of slower visual processing or represent a compensatory strategy to suppress postural movement. The increase in gait COM motion complexity in older adults suggests the dual tasks led to more automatic gait control resulting from both cognitive and visual tasks.

**Supplementary Information:**

The online version contains supplementary material available at 10.1007/s00221-023-06627-4.

## Introduction

Age-related changes in neuromuscular, executive and sensory function impair balance and walking gait stability (Coppin et al. 2006; Aboutorabi et al. 2016; Walsh et al. 2022). The maintenance of balance requires complex integration of sensory information and effective muscle output, the decline in which leads to altered postural control (Walsh et al. 2022). These neuromuscular alterations have also been identified as contributors to age-related changes in spatio-temporal gait characteristics such as step time, stride time or step width and an increase in their variability (Kobsar et al. 2014; Herssens et al. 2018). Changes to the spatio-temporal characteristics and an increased variability of the gait pattern can result in a loss of gait stability and have long been identified as indicators of falls and multiple falls risk (Hausdorff et al. 2001; Callisaya et al. 2011; Lockhart and Liu 2008; Riva et al. 2013). Falls cause significant health, social and economic burden; it is, therefore, essential to understand causes and contributors to gait stability and falls risk and how the balance control systems are impacted by ageing. Ageing and lower muscle function also leads to a loss of gait complexity of gait and postural control, as indicated by the complexity of centre of mass (COM) motion (Bisi and Stagni 2016; Walsh 2021a; Walsh et al. 2022) suggesting less robust postural control. It has also been suggested that a loss of COM motion complexity indicates less automatic postural control of standing and walking (Bisi and Stagni 2016; Richer et al. 2017) indicating older adults invest greater cognitive resources in motor control.

Previous research has demonstrated that older adults utilise a greater internal focus of attention, or movement reinvestment, on the control of movement and balance compared to younger adults, which may increase the cognitive resources required to maintain stability (de Melker Worms et al. 2017; Ellmers et al. 2020). Additionally, dual tasks that increase the demand on the cognitive resources available for processing and integration of information require additional connectivity with motor and executive function regions (Droby et al. 2022). This results in a decrease in walking speed (Smith et al. 2017; Zukowski et al. 2021), increased stride and sway variability (Asai et al. 2019) and alterations to neuromuscular control signals (Walsh 2021b). Changes in cognitive function and demand and the subsequent gait alterations previously reported may manifest in changes to COM motion complexity. These changes would be indicative of the contribution of cognitive resources to gait stability as the progression and stability of the COM is determined by the executed gait pattern.

It has also been demonstrated that dual tasks that constrain gaze behaviour led to alterations in the margins of stability (Kao et al. 2015), greater gait speed variability (Krasovsky et al. 2021) and greater desynchronization in the beta frequency band of the posterior parietal cortex, which is associated with sensory integration during movement (Pizzamiglio et al. 2018). These studies often employ tasks that require concurrent motor activity, e.g., walking while texting (Kao et al. 2015; Pizzamiglio et al. 2018). However, when moving in the real-world gaze behaviour is often constrained or necessarily diverted from a focus on the environmental cues required to walk safely in complex environments without an additional manual task beyond walking. Therefore, insight into the effects of visual dual tasks on gait stability will provide understanding of the interaction of age effects and visual constraint on balance and falls risk.

Recent findings have also demonstrated that gaze complexity and visual intake durations increase during cognitive dual tasks indicating an interaction between regions responsible for cognitive processing such as the prefrontal cortex and the visual cortex (Walsh 2021a). Older adults also demonstrated greater visual input durations during quiet standing than younger adults (Walsh 2021a) and older fallers have longer input durations than older non-fallers (Zukowski et al. 2021). These findings imply that tasks which constrain gaze may have a greater impact on the gait stability of older adults than younger adults.

In the present study, we aimed to determine the effects of age and visual and cognitive dual tasks on gait, assessed using inertial measurement unit (IMU) signals, and gaze behaviour, using wearable, mobile eye-tracking technology. It was hypothesised that (i) older adults would adopt less complex COM motion during gait than younger adults, with longer stride times and greater stride time variability whilst relying on longer visual input durations with correspondingly lower frequency and shorter duration saccades; (ii) that dual task conditions would have greater gait COM motion complexity, longer stride times and greater stride variability, and greater visual input durations with correspondingly lower frequency and duration saccades; (iii) that the effects of dual task on gaze and gait variables would be greater in older than younger adults.

## Methods

### Participants

Twenty participants volunteered for the study, including 10 younger adults and 10 older adults (Table 1). A minimum sample size of 8 participants per group was determined a priori based on an effect size of *f* = 0.53 for the effect of dual tasks on centre of mass postural control (Walsh et al. 2022), a desired power of 0.80 and alpha of 0.05. All participants were free from current lower limb injury, joint replacements, neurological, vestibular and orthopaedic conditions, and had normal or corrected to normal vision. Younger adults were required to be aged between 18 and 35 years and older adults had to be 60 years or older. Participants were explained the purpose of the study, all procedures and their right to withdraw at any time, before providing written informed consent. The study was granted ethical approval by the Oxford Brookes University Research Ethics Committee (registration number: 181196) and all study protocols were conducted in accordance with the Declaration of Helsinki.

**Table 1:** Participant characteristics, mean (standard deviation), for the younger and older adult groups and group differences

Group	Male/Female	Age (yrs)	Height (m)	Mass (kg)	Preferred walking speed (m/s)
Younger adults	7/3	22 (2)	1.76 (0.11)	76.0 (13.5)	4.08 (0.41)
Older adults	5/5	74 (6)	1.73 (0.13)	76.3 (17.2)	3.45 (0.91)
Group difference *p* value	–	–	0.493	0.961	0.091

### Procedures

Participants attended a single laboratory visit during which they performed treadmill (Model PPS 55, Woodway, Wisconsin, USA) walking at their preferred walking speed under a single task control condition (CON), a visual dual task condition (VIS) and a cognitive dual task condition (COG). Prior to commencing the experimental trials participants performed at least 6 min, or until participants reported that they were comfortable and felt they were walking normally, of treadmill familiarisation. Following familiarisation preferred walking speed was determined. With participants blinded to treadmill speed, the speed was increased until participants reported that they were at their normal walking speed, speed was then set above this point and decreased until participants reported that they were at their normal walking speed. The average of the values was used as the preferred walking speed.

For each experimental condition, participants walked at preferred speed for 3 min. Two minutes of seated rest was provided between each trial to reduce the effects of fatigue. The order that conditions were performed was randomised. In all conditions an LCD television screen (1.10 × 0.62 m; Cello Electronics, Durham, UK) was positioned at eye level 2 m in front of the participant when the participant was positioned at the front of the usable treadmill belt area and white screens were placed to each side of the treadmill to minimise visual distractions. During each trial a 9-axis inertial measurement unit (IMU: LPMS-B2, Life Performance Research, Tokyo, Japan) was attached over the L5 vertebrae to measure the movements of the COM, recording at 100 Hz. Participants also wore a pair of mobile eye tracking glasses (Natural Gaze Eye Tracking Glasses, SensoMotoric Instruments, Teltow, Germany) which recorded binocular pupil position within a range of 80° horizontal and 60° vertical to an accuracy of 0.5° at 60 Hz using infrared cameras aimed at each eye.

In CON, participants were given no additional task or instruction as to their gaze behaviour. In VIS, a gaze tracking task was completed. The screen presented a series of line path diagrams (see Supplementary Material for examples of line path diagrams in the format they were presented to participants), e.g., a figure 8 or spiral pattern. Each image was displayed on the screen for 10 s and participants “traced” the line path with their gaze until they reached the end of the path. Each line path had a start point and direction of travel indicated using a red circle and arrow. When participants reached the end of the line path, they were instructed to move their gaze back to the start point immediately and repeat the path. Each line path was repeated for the 10-s period at which point a new image was presented, this cycle was repeated until the end of the 3-min trial. The images presented for the VIS task had a mean (standard deviation) length in their largest dimension of 0.60 m (0.07 m) and visual angle of 17.2° (2.2°). Participants were not instructed on the speed required to complete the visual task but were asked to try to keep their gaze moving continuously along the path during the trial periods, except for when they had to return to the start point, and to follow the path as closely as possible without cutting corners. Adherence with the task was monitored in real time by a member of the research team who was able to track the participants gaze location in the scene by means of the gaze vector superimposed on a scene image recorded by a forward-facing camera integrated in the eye tracking glasses.

In COG, participants were required to count aloud backwards by 7s from a randomly generated 3-digit number. The 3-digit number was displayed on the screen (font size 100 pt, 0.16 × 0.08 m) for 2 s after which the screen returned to white, a new number was presented every 60 s.

### Gait analysis

All IMU gait data were analysed in MATLAB (R2016b, Mathworks Inc., Natick MA, USA) scripts. The acceleration signals from the IMU were used for analysis of gait COM motion complexity and spatio-temporal parameters. Initial contact heel strike events were determined from the anterior–posterior (AP) acceleration of the IMU. The AP acceleration signal was filtered twice separately with 20 and 2 Hz cut-off frequencies using second order dual-pass (fourth order total) Butterworth filters. Initial contacts were determined as the points of the peaks in the 20 Hz filtered signal which immediately precede positive to negative zero-crossings in the 2 Hz filtered signal (McCamley et al. 2012). The middle 120 gait cycles were separated for the AP, medio-lateral (ML) and vertical (VT) axis acceleration signals. The average stride time (ST_MEAN_) was determined as the average time between subsequent ipsilateral heel strikes. The stride time variability (ST_VAR_) was determined as the standard deviation of the stride time.

To determine gait COM motion complexity the refined composite multiscale entropy (RCMSE) was determined as previously described (Wu et al. 2014), using input parameters of *m* = 2 and *r* = 0.2 and τ ranged from 1 to 30 data points. A τ of 30 represents a scale of 300 ms for the IMU signals, which is representative of supraspinal delays (Frost et al. 2015). The complexity index was used to quantify the degree of complexity for each direction (CI_AP_, CI_ML_, CI_VT_) by calculating the area under the curve of the plot of sample entropy vs. τ. A larger complexity index indicates a more complex signal. RCMSE was used instead of traditional multiscale entropy algorithm as it resolves issues related to short signal lengths at higher scale levels and undefined entropy calculations at these scales (Wu et al. 2014; Raffalt et al. 2018).

### Gaze analysis

Gaze data were analysed using BeGaze software (SensoMotoric Instruments, Teltow, Germany). The first and last 2 s of data of each trial were discarded before analysis. For each trial fixations and saccades were identified from the binocular gaze vector time series, determined as the resultant of the horizontal and vertical gaze position. Saccades were defined as periods of eye rotation that exceeded 100◦/s and fixations were defined as periods of at least 50 ms bordered immediately before and after by a saccade (Paquette and Fung 2011). The frequency of saccades (SACC_FREQ_) was determined as the number of saccades per second and the duration of saccades (SA_CCDUR_) as the average duration of all saccades in a trial. The visual input frequency (VISIN_FREQ_) was determined as the number of fixations per second and the visual input duration (VISIN_DUR_) was determined as the average duration of all fixations in a trial.

### Statistics

All statistical analysis was performed using SPSS (v28, IBM Corp., NY, USA). Data were assessed for normality using the Shapiro–Wilk test. Participant height, mass and preferred walking speed were compared between age groups using independent samples t-tests (Table 1). Two separate 2 (younger vs. older) × 3 (CON vs. VIS vs. COG) MANOVA were performed to determine the effect of age and dual task condition and their interaction on gait and gaze variables using the Wilk’s Lambda (λ) test statistic (hypotheses i–iii). For significant multivariate main and interaction effects univariate 3 × 2 ANOVA were performed. For significant univariate main effects of task condition pairwise comparisons with a Bonferroni correction were performed and for significant interaction effects simple main effects with a Bonferroni correction were performed. When task conditions and interactions violated the assumption of sphericity, the Greenhouse–Geisser correction to the degrees of freedom was applied. For the MANOVA and subsequent ANOVA partial eta squared (${\eta }_{p}^{2}$) effect size was calculated, values of 0.01, 0.06 and 0.14 represent small, moderate and large effects, respectively (Cohen 1988; Richardson 2011). For all tests, the level of significance was *p* < 0.05.

## Results

### Dual task adherence

All participants in both groups adhered to the cognitive dual tasks for the duration of each trial. All younger adults adhered to the visual task without the need to repeat trials. Two older adults were required to repeat a total of three visual task trials as they did not appropriately track the patterns required.

### Effects of age and dual task on gait

**Fig. 1:**
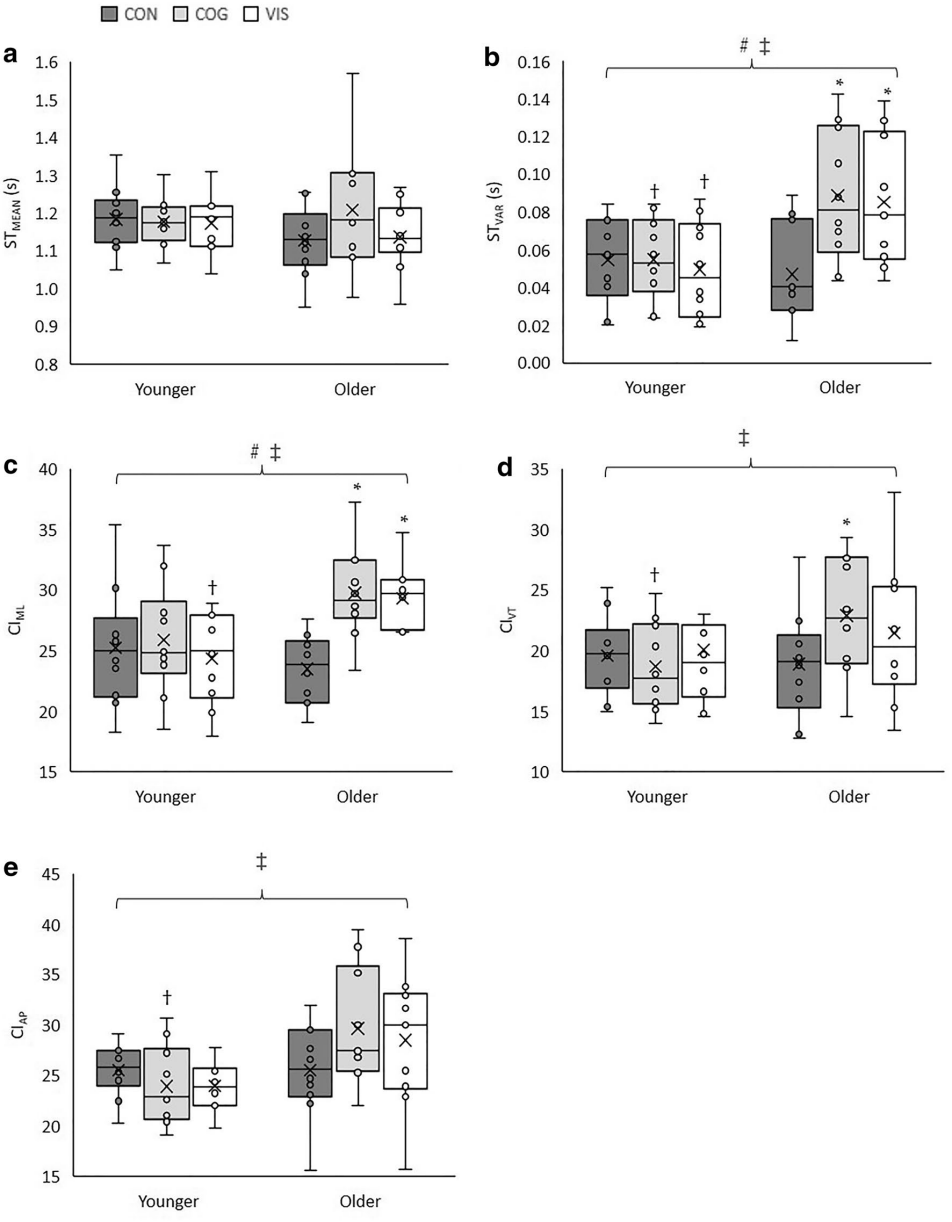
Boxplots including individual data points for **a** ST_MEAN_, **b** ST_VAR_, **c** CI_ML_, **d** CI_VT_ and **e** CI_AP_. The mean of each condition is represented by an X. The upper, middle and bottom horizontal line of each box represent the 1st quartile, median and 3rd quartile of the data, respectively, and the error bars indicate the minimum and maximum values. † Indicates value is lower in younger adults than older adults. *Value is greater than CON. #A main effect of dual task condition. ‡An interaction effect

There were significant multivariate effects of age group (λ = 0.46, *F*(5,14) = 3.30, *p* = 0.035, ${\eta }_{p}^{2}$=0.54), dual task condition (λ = 0.44, *F*(10,64) = 3.20, *p* = 0.002, ${\eta }_{p}^{2}$=0.33) and group x task interaction (λ = 0.42, *F*(10,64) = 3.45, *p* = 0.001, ${\eta }_{p}^{2}$=0.35). Data for gait variables are shown in Fig. 1.

There were no significant univariate main effects of age group for any gait variable. For dual task conditions there was a significant main effect for ST_VAR_ (*F*(1.48,26.65) = 4.63, *p* = 0.028, ${\eta }_{p}^{2}$=0.21) and CI_ML_ (*F*(2,36) = 7.24, *p* = 0.002, ${\eta }_{p}^{2}$=0.29). There were no significant effects of dual task for any other gait variable. For ST_VAR_ no pairwise comparison were significant following correction for multiple comparisons. CI_ML_ was greater for COG than CON (*p* = 0.004).

The age group x dual task condition interaction was significant for ST_VAR_ (F(1.48,26.65) = 6.15, *p* = 0.011, ${\eta }_{p}^{2}$=0.26), CI_ML_ (*F*(2,36) = 7.40, *p* = 0.002, ${\eta }_{p}^{2}$=0.29), CI_AP_ (*F*(2,36) = 3.96, *p* = 0.028, ${\eta }_{p}^{2}$=0.18) and CI_VT_ (*F*(2,36) = 3.79, *p* = 0.032, ${\eta }_{p}^{2}$=0.17). Analysis of simple main effects found that there was no difference between conditions for any variable in younger adults. However, COG was greater than CON in older adults for ST_VAR_ (*p* = 0.006), CI_ML_ (*p* < 0.001) and CI_VT_ (*p* = 0.007) and VIS was greater than CON in older adults for ST_VAR_ (*p* = 0.001) and CI_ML_ (*p* = 0.003). Additionally, in COG older adults were greater than younger for ST_VAR_ (*p* = 0.019), CI_VT_ (*p* = 0.044) and CI_AP_ (*p* = 0.019) and in VIS older adults were greater in ST_VAR_ (*p* = 0.020) and CI_ML_ (*p* = 0.003).

### Effects of age and dual task on gaze

**Fig. 2:**
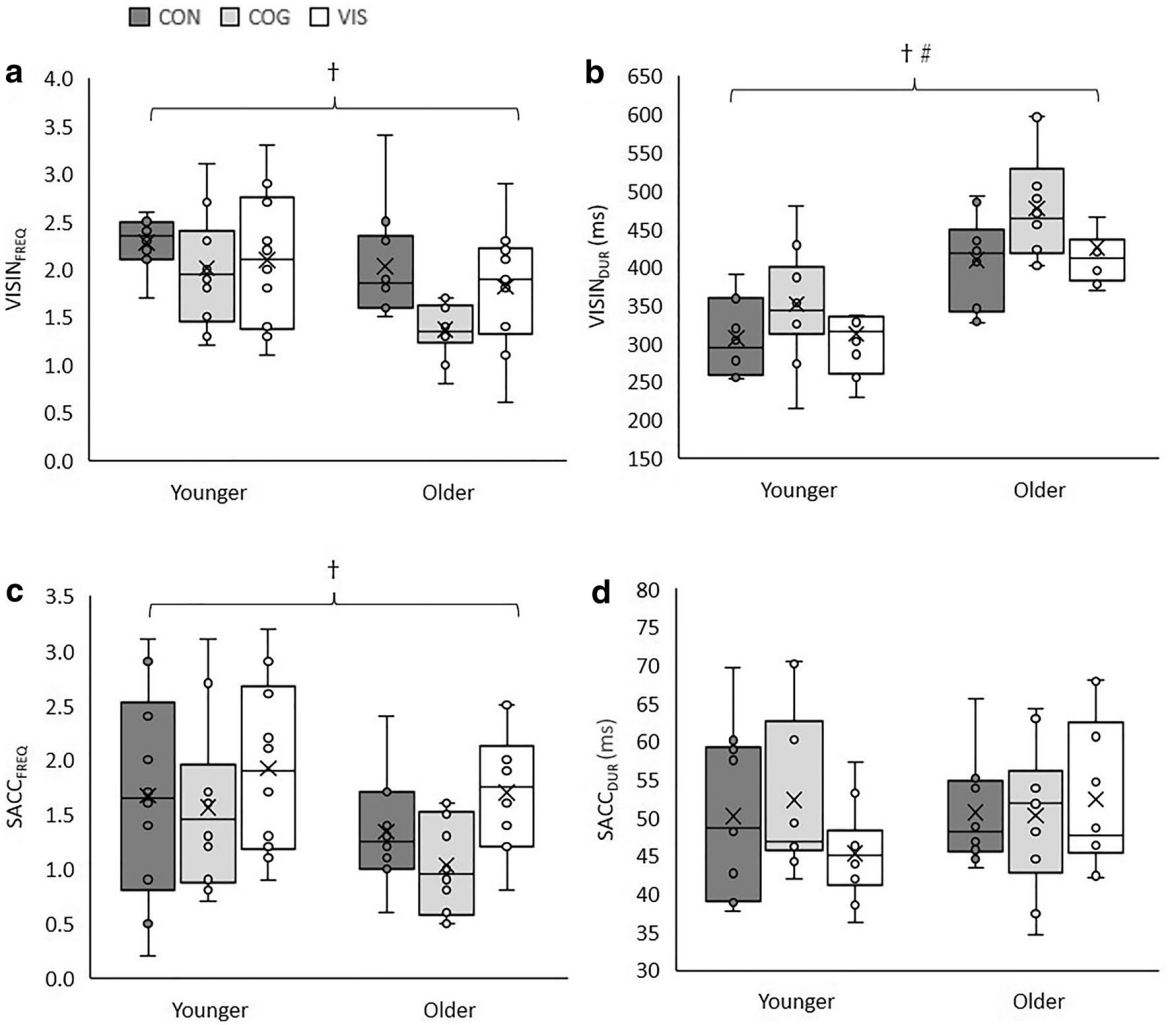
Boxplots including individual data points for **a** VISIN_FREQ_, **b** VISIN_DUR_, **c** SACC_FREQ_ and **d** SACC_DUR_. The mean of each condition is represented by an X. The upper, middle and bottom horizontal line of each box represent the 1st quartile, median and 3rd quartile of the data, respectively, and the error bars indicate the minimum and maximum values. †A main effect of age group. #A main effect of dual task condition but no pairwise differences

There were significant multivariate effects of age group (λ = 0.26, *F*(4,15) = 10.64, *p* < 0.001, ${\eta }_{p}^{2}$=0.74) and dual task condition (λ = 0.62, *F*(8,66) = 2.22, *p* = 0.037, ${\eta }_{p}^{2}$=0.21) but no group x task interaction (λ = 0.84, *F*(8,66) = 0.77, *p* = 0.631, ${\eta }_{p}^{2}$=0.09). Data for gaze variables are shown in Fig. 2.

Significant effects of age group were present for VISIN_FREQ_ (*F*(1,18) = 10.98, *p* = 0.004, ${\eta }_{p}^{2}$=0.38), VISIN_DUR_ (*F*(1,18) = 43.13, *p* < 0.001, ${\eta }_{p}^{2}$=0.71) and SACC_FREQ_ (*F*(1,18) = 6.69, *p* = 0.019, ${\eta }_{p}^{2}$=0.27). VISIN_FREQ_ and SACC_FREQ_ were greater in younger compared to older adults and VIS_DUR_ was greater in older compared to younger adults.

There was a significant effect of task on VISIN_DUR_ (*F*(2,36) = 4.33, *p* = 0.021, ${\eta }_{p}^{2}$=0.19) but no significant effect for any other gaze variable. For VISIN_DUR_ no pairwise comparisons were significant following correction for multiple comparisons.

## Discussion

The purpose of this study was to determine the effects of age and visual and cognitive dual tasks on gait and gaze behaviour. Hypothesis (i) was partially accepted as analysis of simple main effects found that older adults had greater stride time variability in the dual task conditions, longer visual input durations and lower saccade frequency irrespective of dual task condition but did not have lower gait COM motion complexity or mean stride times compared to younger adults. Similarly, hypothesis (ii) was partially accepted as dual task conditions resulted in greater stride time variability, more complex COM motion and variable stride times but had no effect on gaze behaviour or mean stride time. Finally, hypothesis (iii) was also partially accepted as there were significant interaction effects for gait variables with older adults demonstrating greater effects on gait than younger adults, but there were no significant interaction effects for gaze variables.

Analysis of interaction effects in this study found that in older adults both COG and VIS increased stride time variability and COM motion complexity compared to CON, in agreement with previous findings reporting an increase in gait and standing centre of mass motion complexity during dual tasks (Stins et al. 2009; Richer and Lajoie 2020; Walsh 2021b, a). An increase in gait COM motion complexity is indicative of more autonomous execution of gait when cognitive resources for gait control are directed to a concurrent task (Bisi and Stagni 2016; Richer and Lajoie 2020). Contrary to the hypothesised effect, there was no main effect of age group on stride time mean or gait COM motion complexity. This may be explained by the otherwise healthy, active older adult population recruited, although the significant interaction effects resulting from changes in older adults not present in younger adults was as hypothesised. The lack of change in younger adults may indicate that the tasks utilised in this study were not sufficiently challenging to lead to measurable changes in gait COM motion complexity, stride time or stride time variability. The interaction effects found could also be explained by the greater movement reinvestment reported in older adults (de Melker Worms et al. 2017; Ellmers et al. 2020) which requires significant cognitive resource dedicated to movement control, when this is disrupted by a dual task it may be expected for a greater effect to be present in older adults than younger.

As with ML COM motion complexity, analysis of significant interaction effects revealed that stride time variability was significantly greater in older adults during dual task conditions compared to CON and was greater in older adults during the dual task conditions compared to younger adults. These findings are in line with the hypothesised effects and suggest that the gait pattern of older adults was less consistent or precisely controlled (Almarwani et al. 2016) during both dual tasks and when compared to younger adults when performing these tasks. Interestingly, contrary to our hypotheses there were no effects of age group or dual task on stride time mean. An explanation for the unchanged stride time mean despite the stride time variability changing may be the result of the use of a treadmill in this study. The treadmill requires participants to maintain a constant speed, so whilst stride time fluctuated in older adults during dual tasks (increased stride time variability) the stride time mean could not be allowed to decrease significantly to ensure participants were able to match the treadmill speed.

The increase in gait COM motion complexity in older adults in VIS suggest that tasks that solely constrain gaze behaviour and do not include additional cognitive or motor tasks, unlike tasks such as texting, also significantly altered gait COM motion complexity increasing the automaticity of gait control, albeit only in the ML direction. This finding agrees with previous literature demonstrating the effect of visually demanding tasks on gait in older adults (Beurskens and Bock 2013) and on the role of visual information in balance control (Walsh 2021a). However, this response was only seen in the ML direction in the present study and not in the AP or VT directions which may be explained by the ability of the passive dynamics of gait to control stability in these directions, whereas the ML direction requires active control to maintain stability (Bruijn and Van Dieën 2018; Reimann et al. 2018). The COG task increased COM motion complexity in both the ML and VT, which suggests that this condition had a broader effect on gait in older adults than VIS, with the lack of change in the AP again explained by the role of passive dynamics in gait control of this direction. The differing response to the COG and VIS tasks could be explained by the use of treadmill locomotion in this study. The treadmill provides a predictable environment to move in that may reduce the needed for regular visual sensory input regarding the state of the environment and placement of feet as the risk of collision or tripping is low. Therefore, the effect of a visual restraint task on gait dynamics is relatively smaller than that of a cognitively demanding task.

The present study found that older adults relied on longer visual input duration compared to younger adults as has been found previously during standing tasks (Walsh 2021a). Accordingly, in this study older adults also had lower visual input frequency and saccade frequency. These findings could be indicative of the greater time required to process visual information in older adults compared to younger adults (Ebaid and Crewther 2019). However, during standing tasks the suppression of eye movements with longer duration fixations reduces postural sway (Jahn et al. 2002). An alternative interpretation of these findings is therefore that older adults adopted a similar compensation during walking to control centre of mass motion as a compensatory strategy. Contrary to our hypothesis and the changes in saccade frequency and visual input parameters, there were no effects on saccade duration. This suggests that although older adults adopt longer visual inputs, the speed at which saccades are performed is not altered which was accommodated for by decreasing visual input frequency and saccade frequency. This is in agreement with previous findings which have demonstrated saccade speed to be relatively invariant in older adults during all but the most challenging gaze tasks (Bae 2022).

In opposition to previous research and the hypothesised effect (Walsh 2021a), the dual task conditions had no effect on gaze behaviour. There was a significant main effect of task on visual input duration_,_ which would be in agreement with previous findings, however, no pairwise comparisons survived correction for multiple comparisons. It is possible that the walking task employed in the current study represented a more challenging motor task than the quiet standing paradigm investigated previously (Walsh 2021a) and, therefore, participants minimised disruption to the sensory information provided by visual input. Similarly, it has been demonstrated in a walking task requiring participants to avoid collisions that dual task effects were not seen in gait or gaze parameters, indicating participants prioritized the locomotor task at hand (Bhojwani et al. 2022).

There were limitations of the present study that should be considered. First, the study recruited only healthy community-dwelling older adults, therefore these findings may not be applicable to higher fall risk older adults, for example those with significant frailty, comorbidities or those in full-time care facilities. Additionally, whilst the use of treadmill walking allows for the analysis of longer durations of walking it may not be representative of real-world walking conditions, where task difficulty is greater and there may be a range of concurrent cognitive and visual tasks. The relationship between gaze and gait behaviour in postural control while walking in real-world complex environments warrants further investigation. It should also be considered that the performance of the secondary dual tasks was not objectively quantified in this study. As a result, some caution should be taken when interpreting the dual task effects reported as it is not possible to differentiate whether the groups prioritised tasks similarly. However, this does not prevent an objective global assessment of the effects of dual tasks, as was the a priori intention of this study, but does limit the understanding of the underlying mechanisms specific to how cognitive resources are allocated. Finally, the use of screens to prevent unexpected movements or distractions in the laboratory and help task adherence may have impacted peripheral visual stimuli of participants. It is possible that this may have had a greater impact on older adults.

In conclusion, this study has demonstrated that both cognitive and visual dual tasks alter gait dynamics of older adults but had limited effect on visual behaviour. Both dual task conditions led to greater complexity of centre of mass motion and stride time variability in older adults, indicative of more automatic gait control. However, there were no effects on younger adult’s gait suggesting that the dual task conditions adopted were insufficiently challenging to elicit changes in this population. Older adults relied on longer visual input durations with less frequent fixations and saccades than younger adults, which may be a compensatory strategy for slower visual information processing or to control centre of mass motion.

## Supplementary Information

Below is the link to the electronic supplementary material.

Supplementary file1 (PPTX 69 KB)

## Data Availability

The data sets analysed during the current study are available from the corresponding author on reasonable request.
