# Supplementary material for: Cognitive and visual task effects on gaze behaviour and gait of younger and older adults
Source: Exp Brain Res. 2023 May 6;241(6):1623–31. doi: 10.1007/s00221-023-06627-4 (PMC10224856; doi:10.1007/s00221-023-06627-4)

## Slide 1
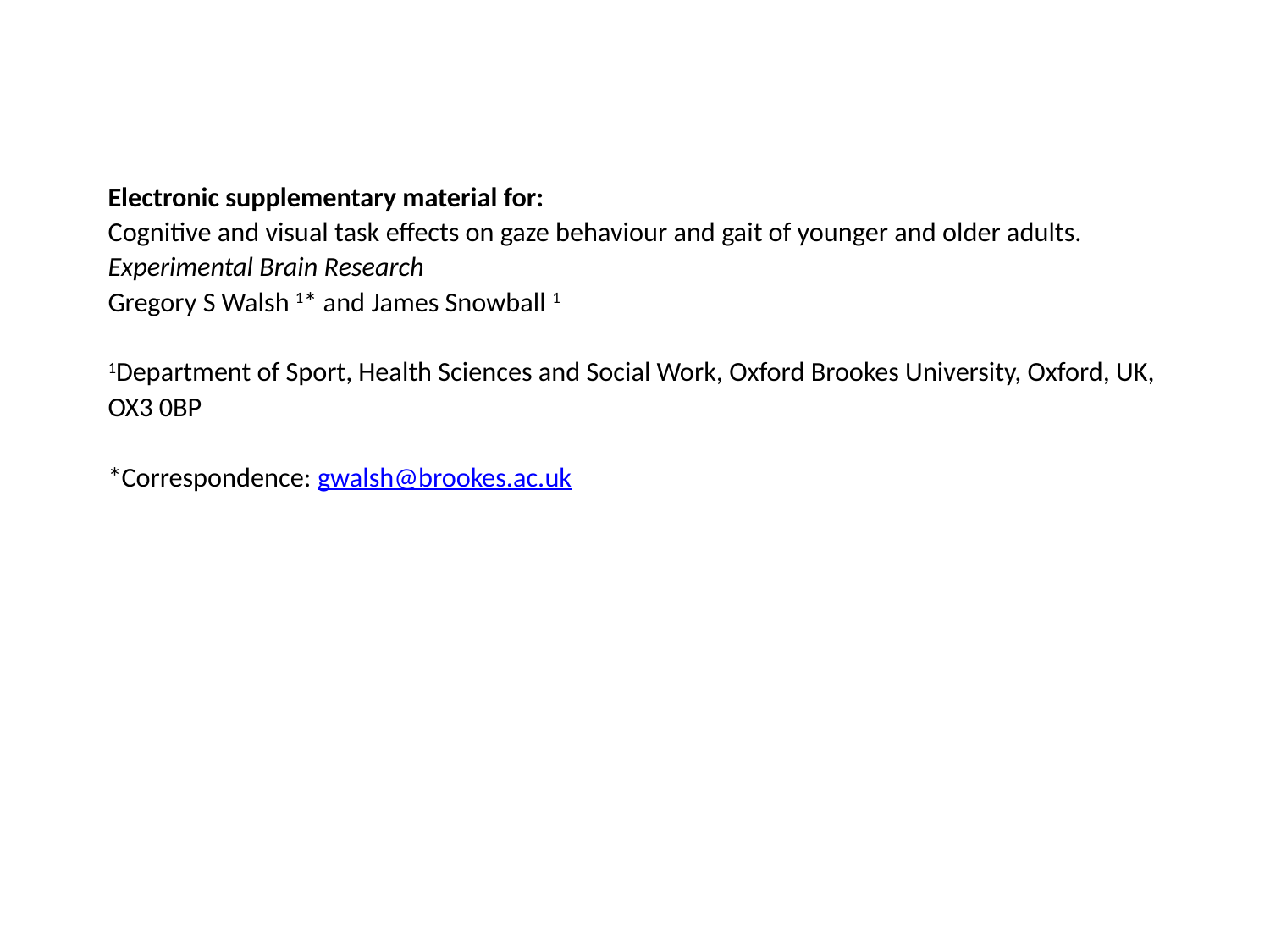

# Electronic supplementary material for: Cognitive and visual task effects on gaze behaviour and gait of younger and older adults. Experimental Brain Research Gregory S Walsh 1* and James Snowball 1   1Department of Sport, Health Sciences and Social Work, Oxford Brookes University, Oxford, UK, OX3 0BP *Correspondence: gwalsh@brookes.ac.uk

## Slide 2
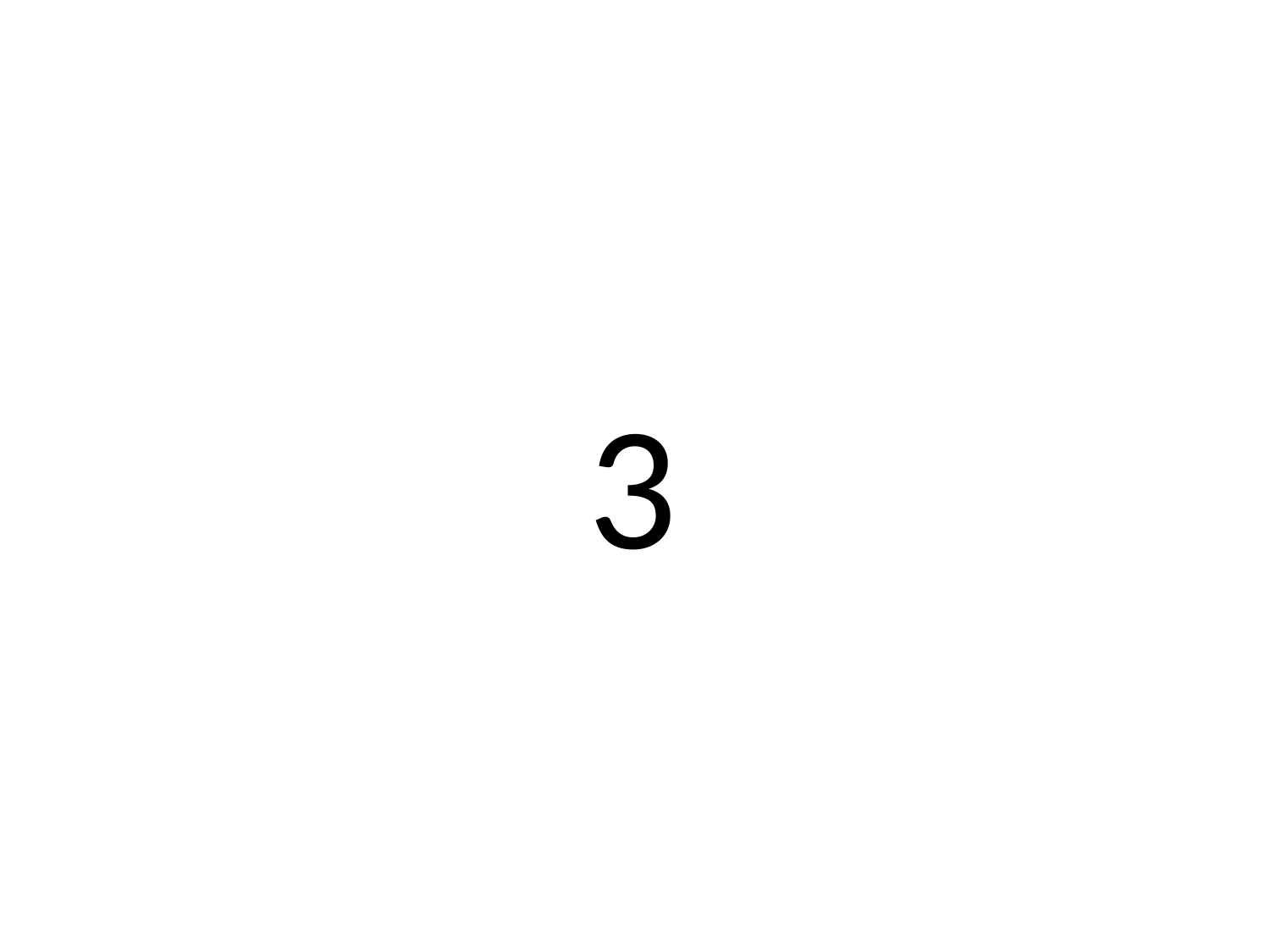

#
3

## Slide 3
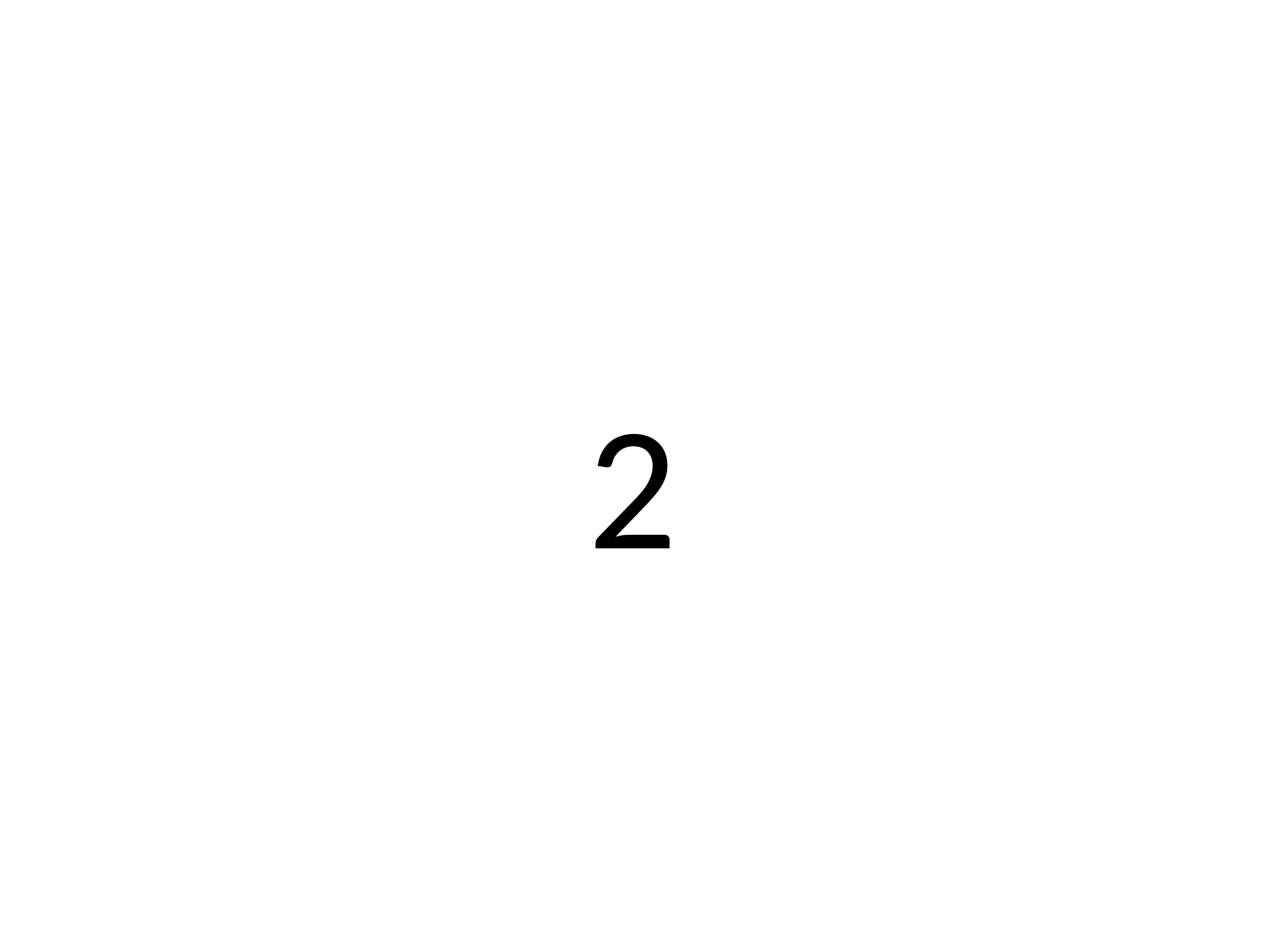

#
2

## Slide 4
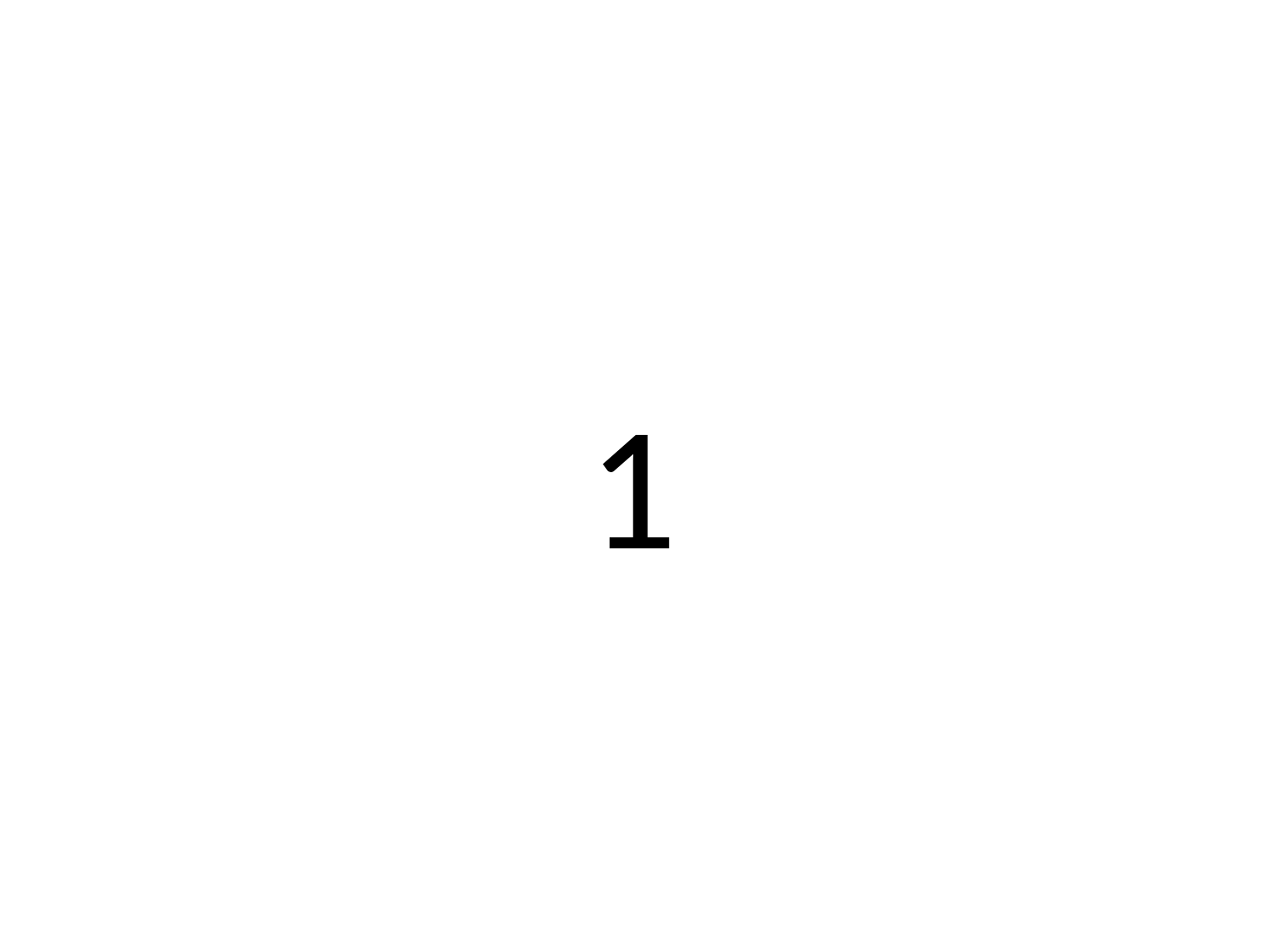

#
1

## Slide 5
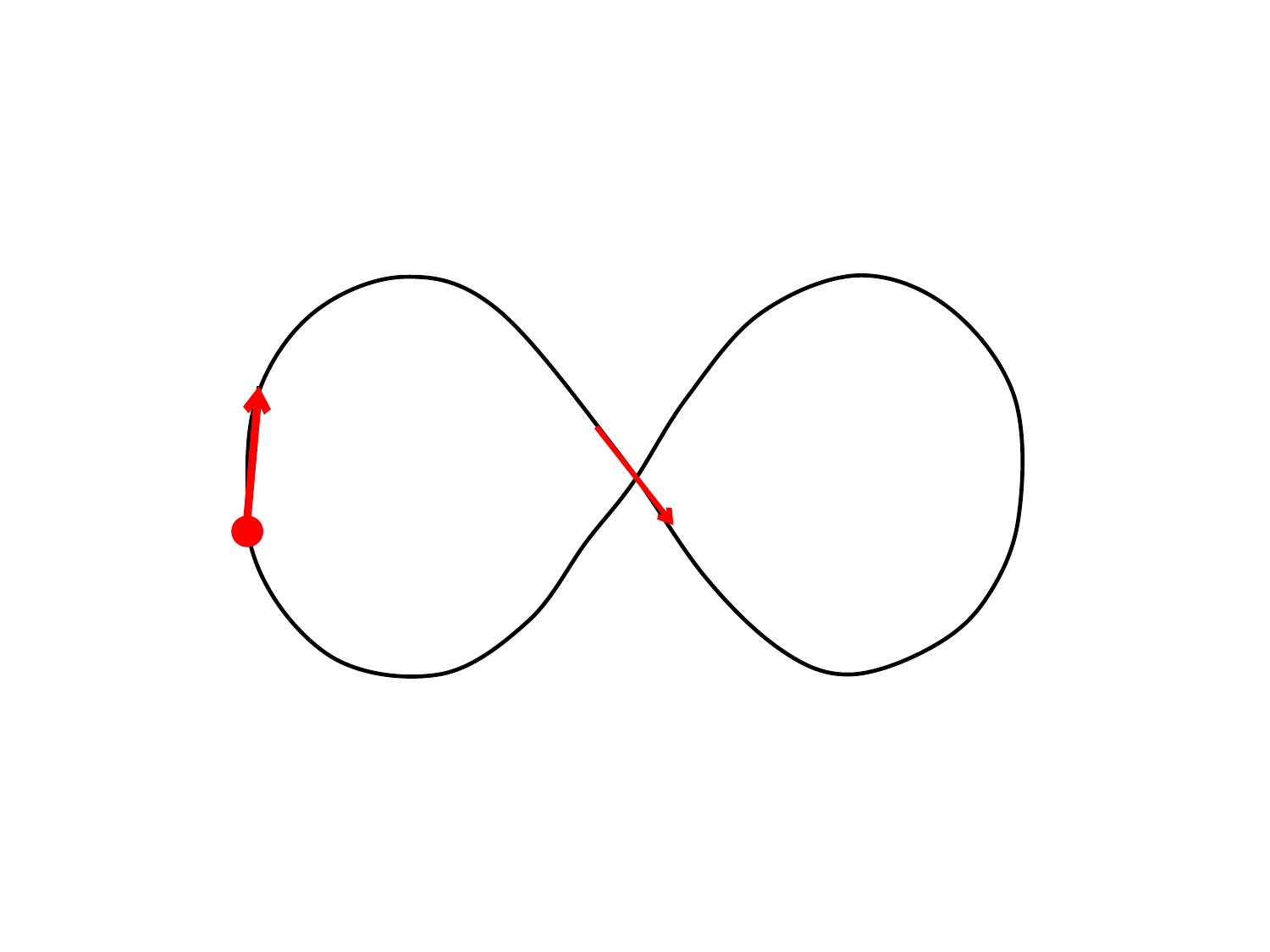

## Slide 6
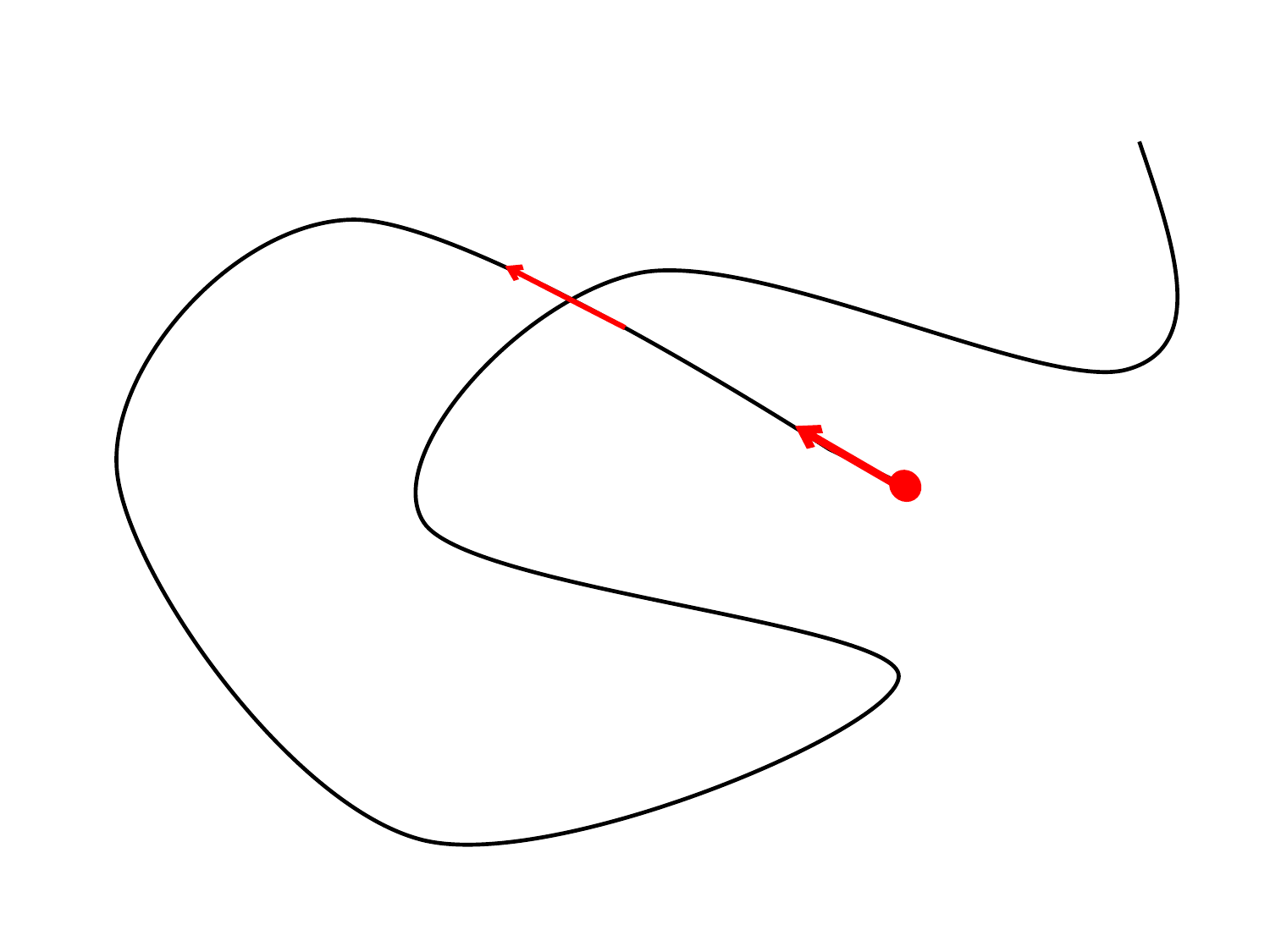

## Slide 7
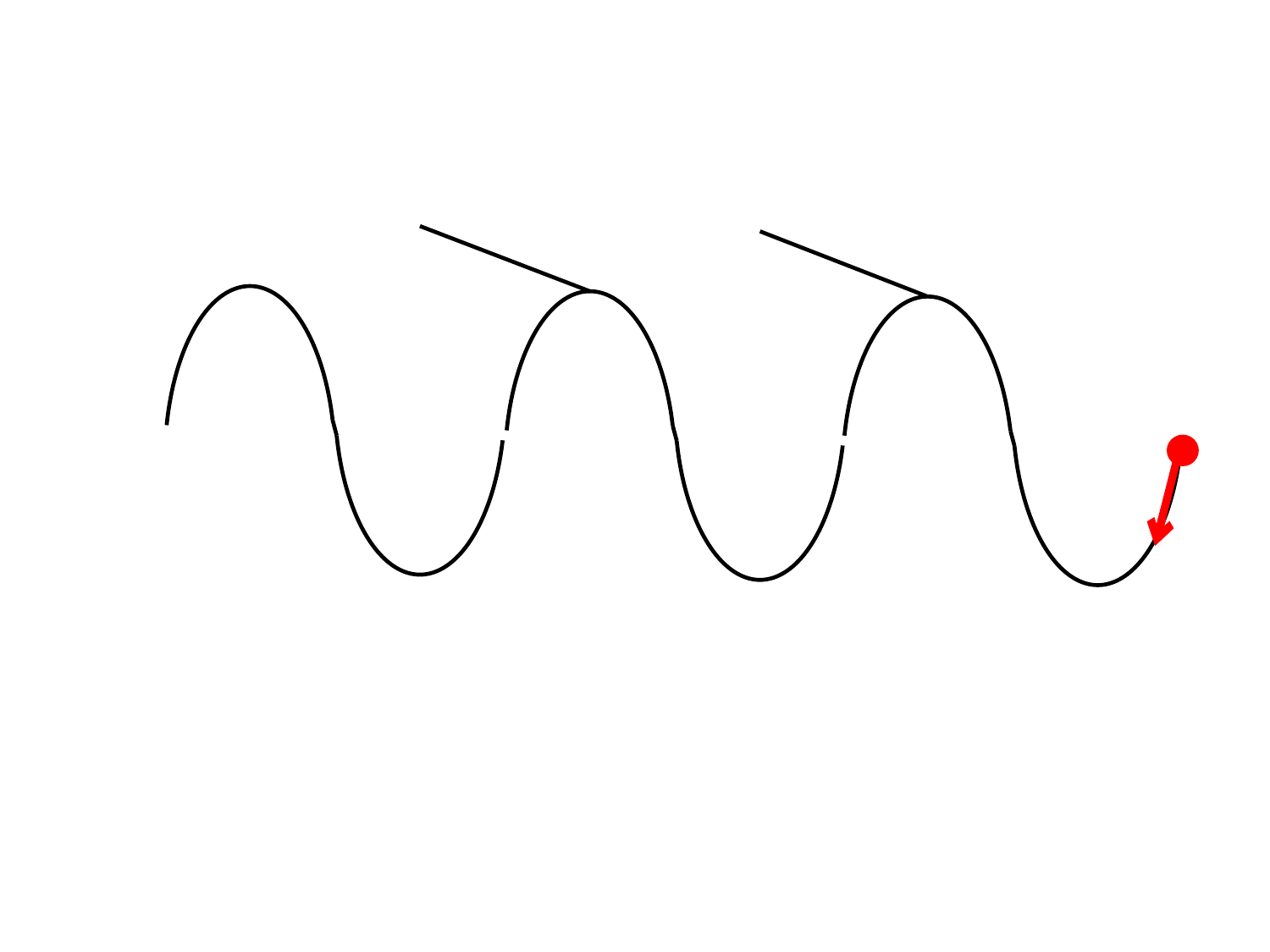

## Slide 8
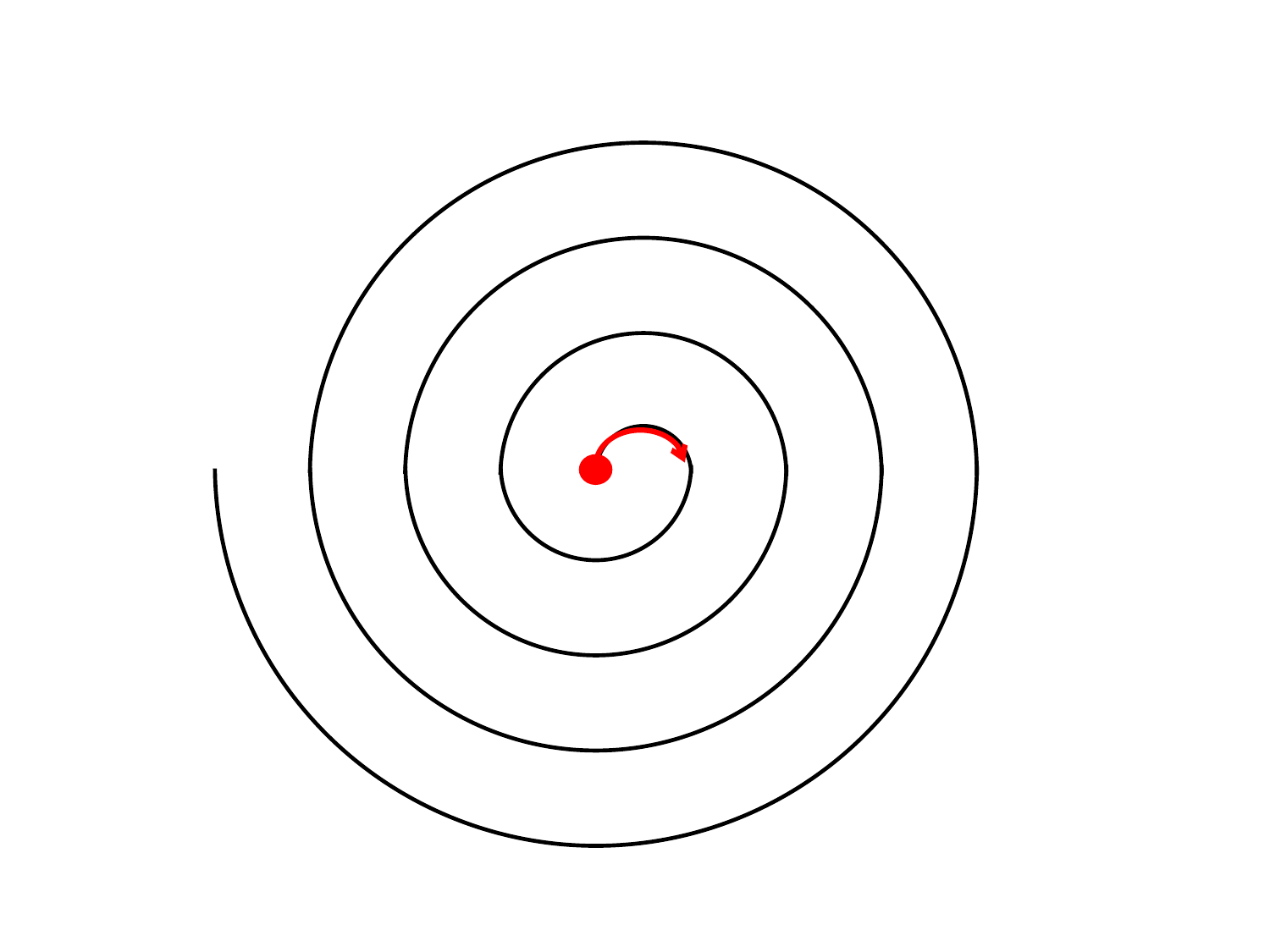

## Slide 9
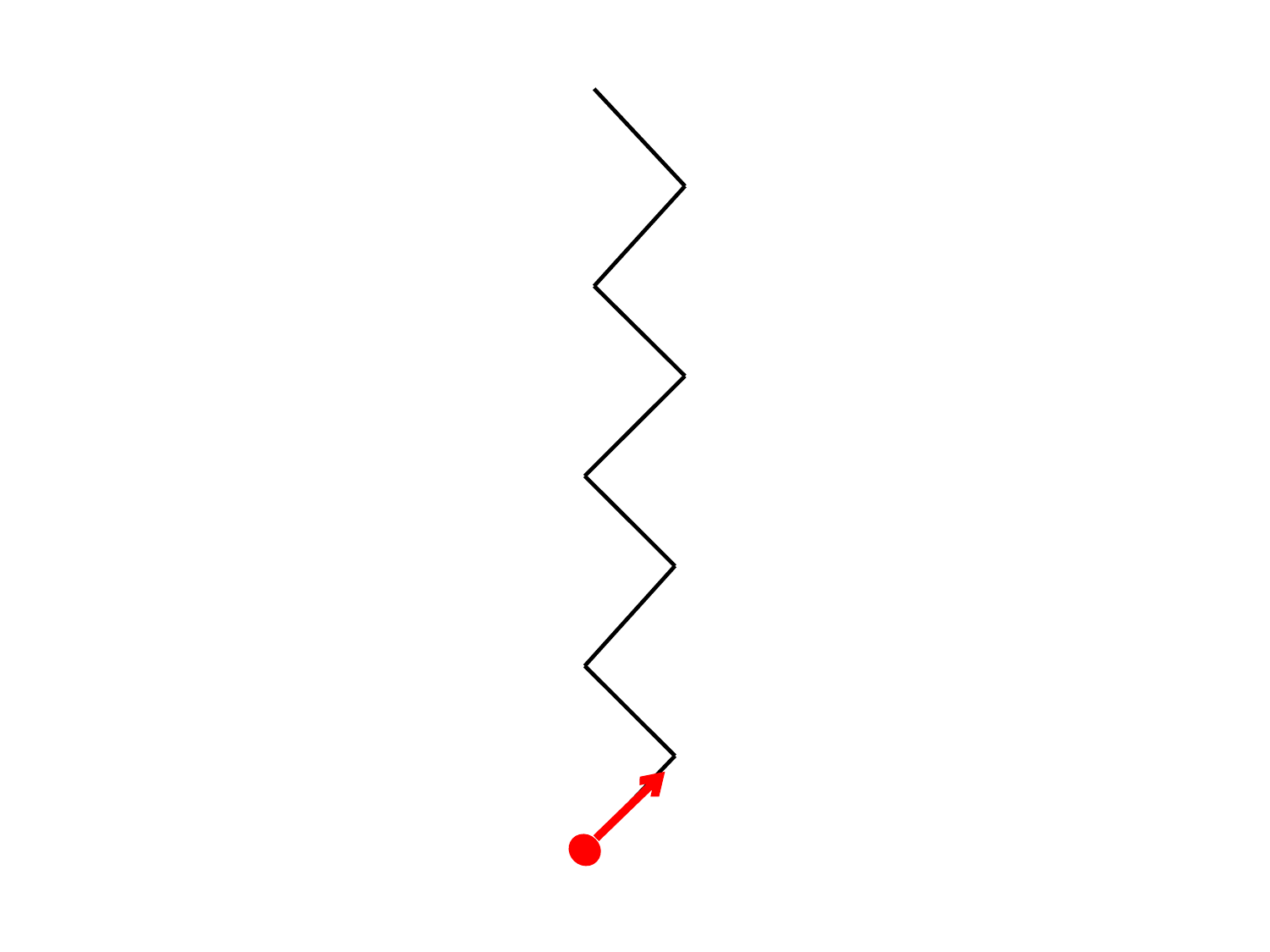

## Slide 10
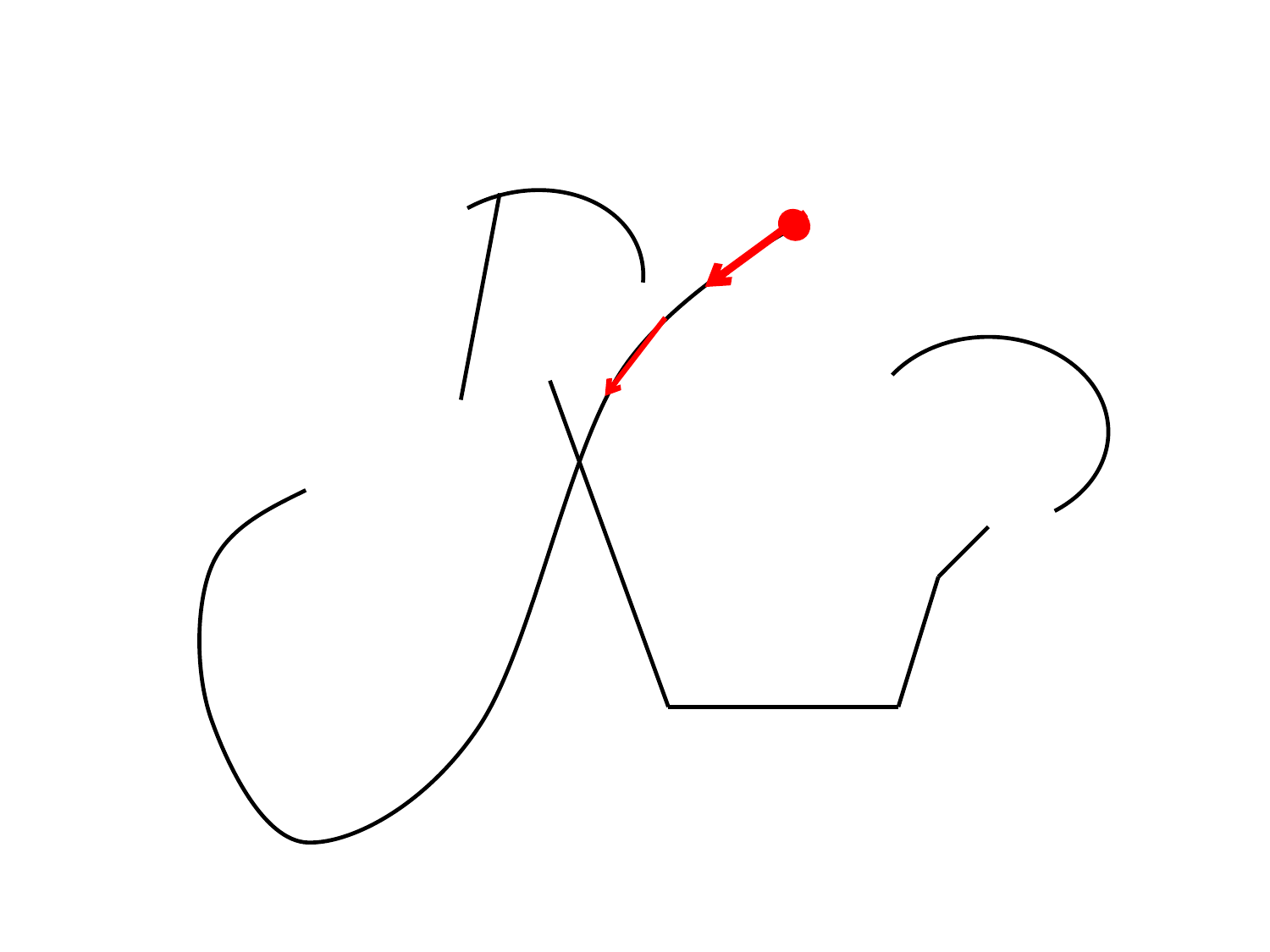

## Slide 11
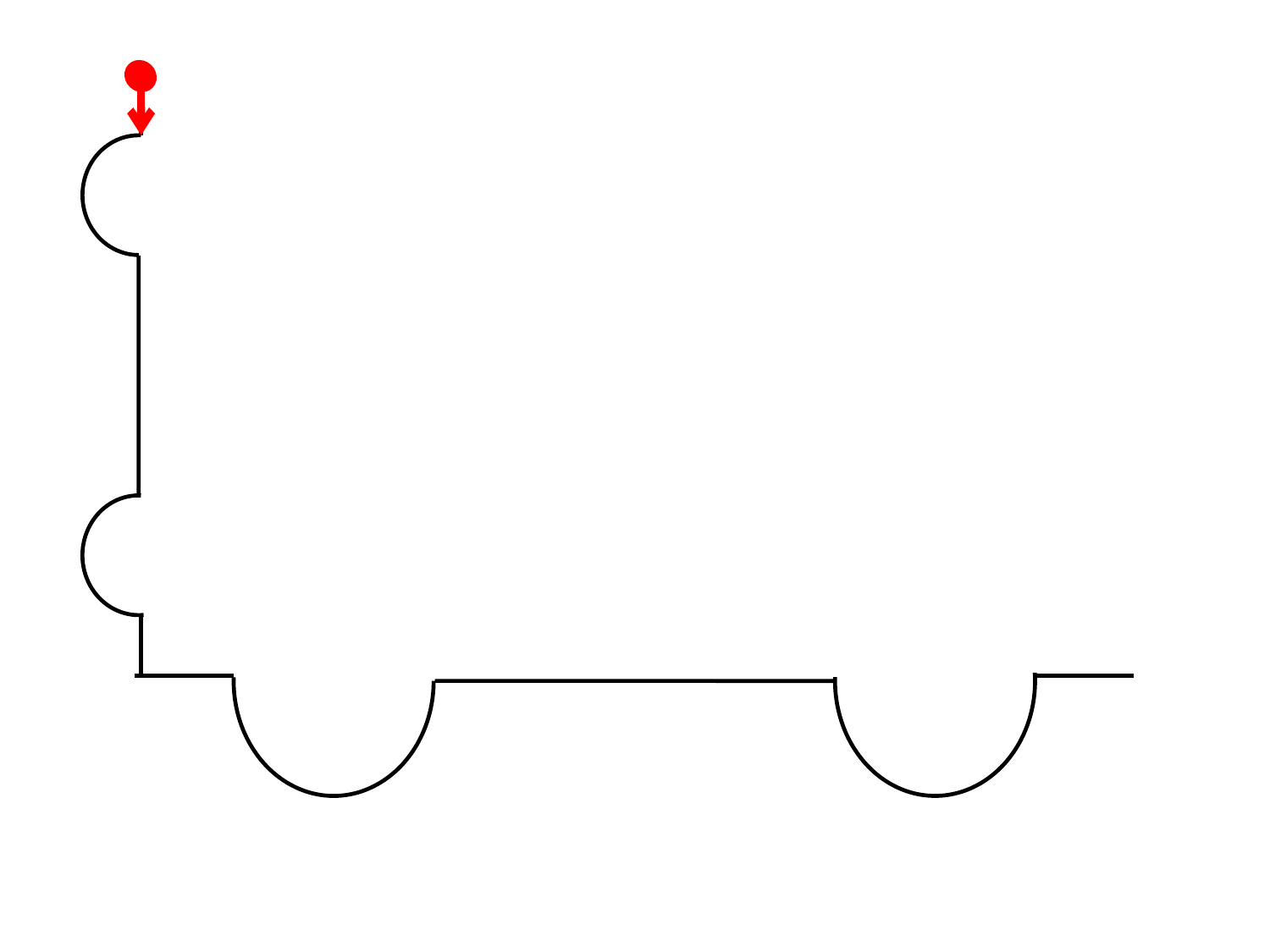

## Slide 12
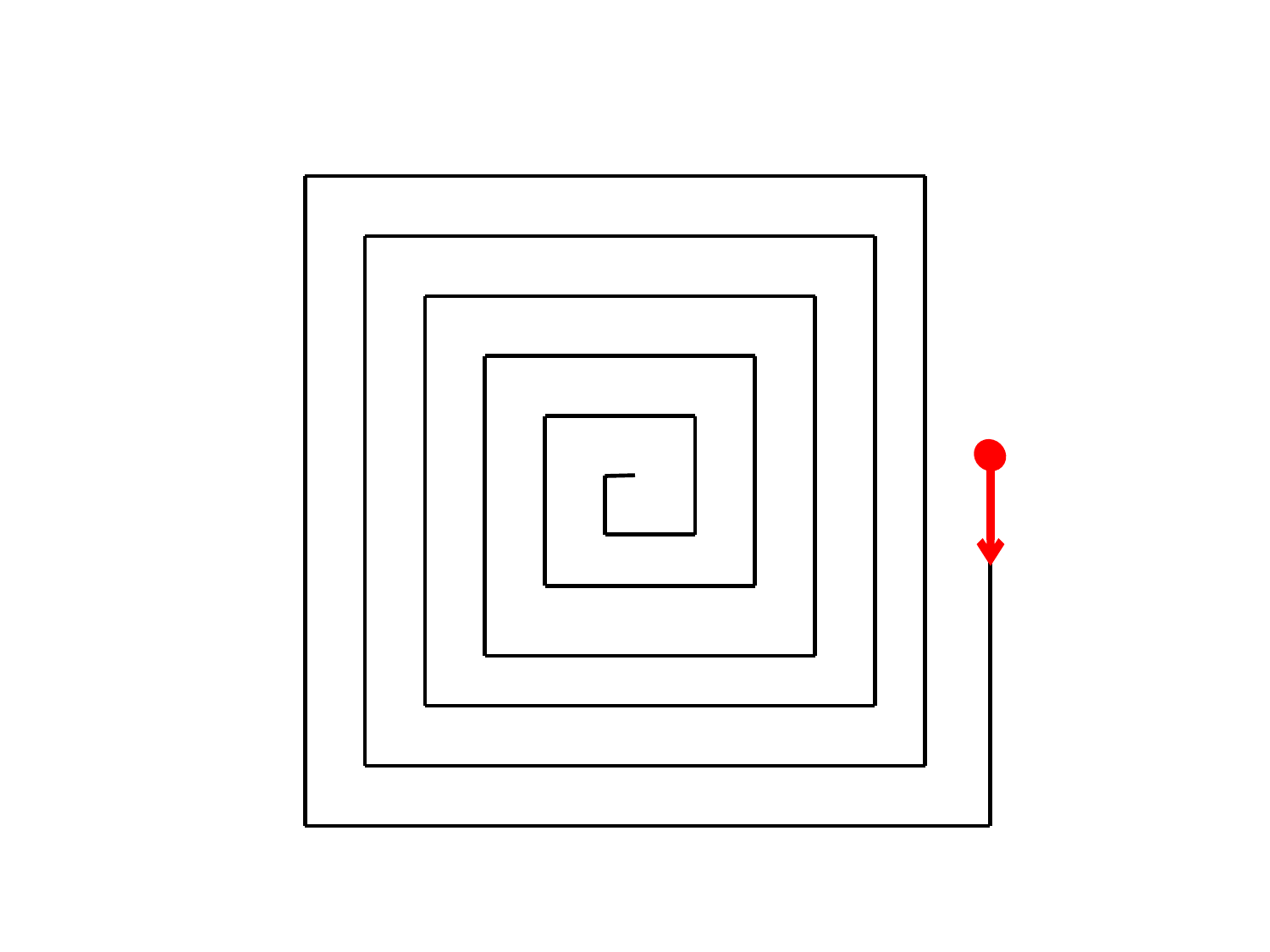

## Slide 13
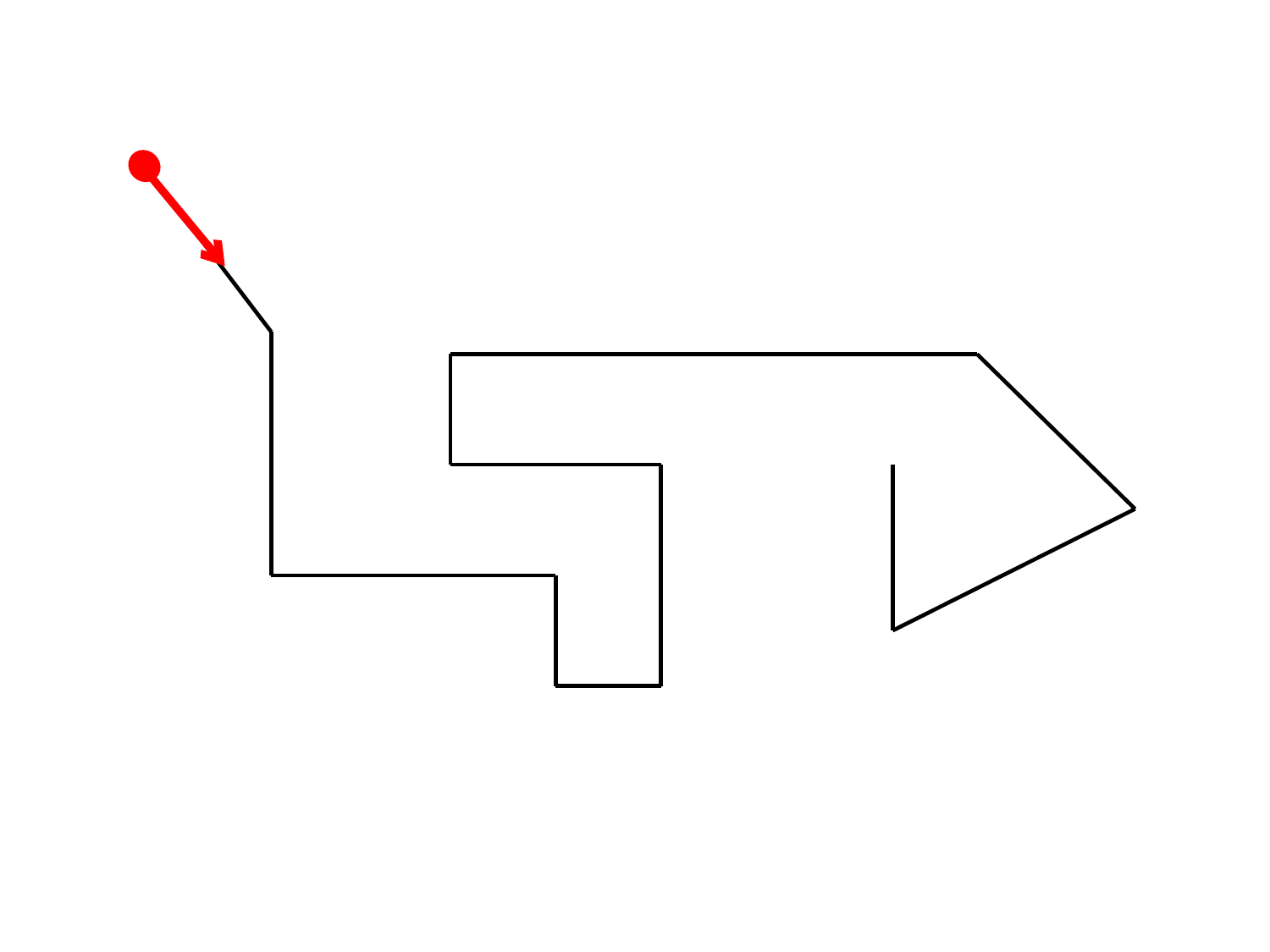

## Slide 14
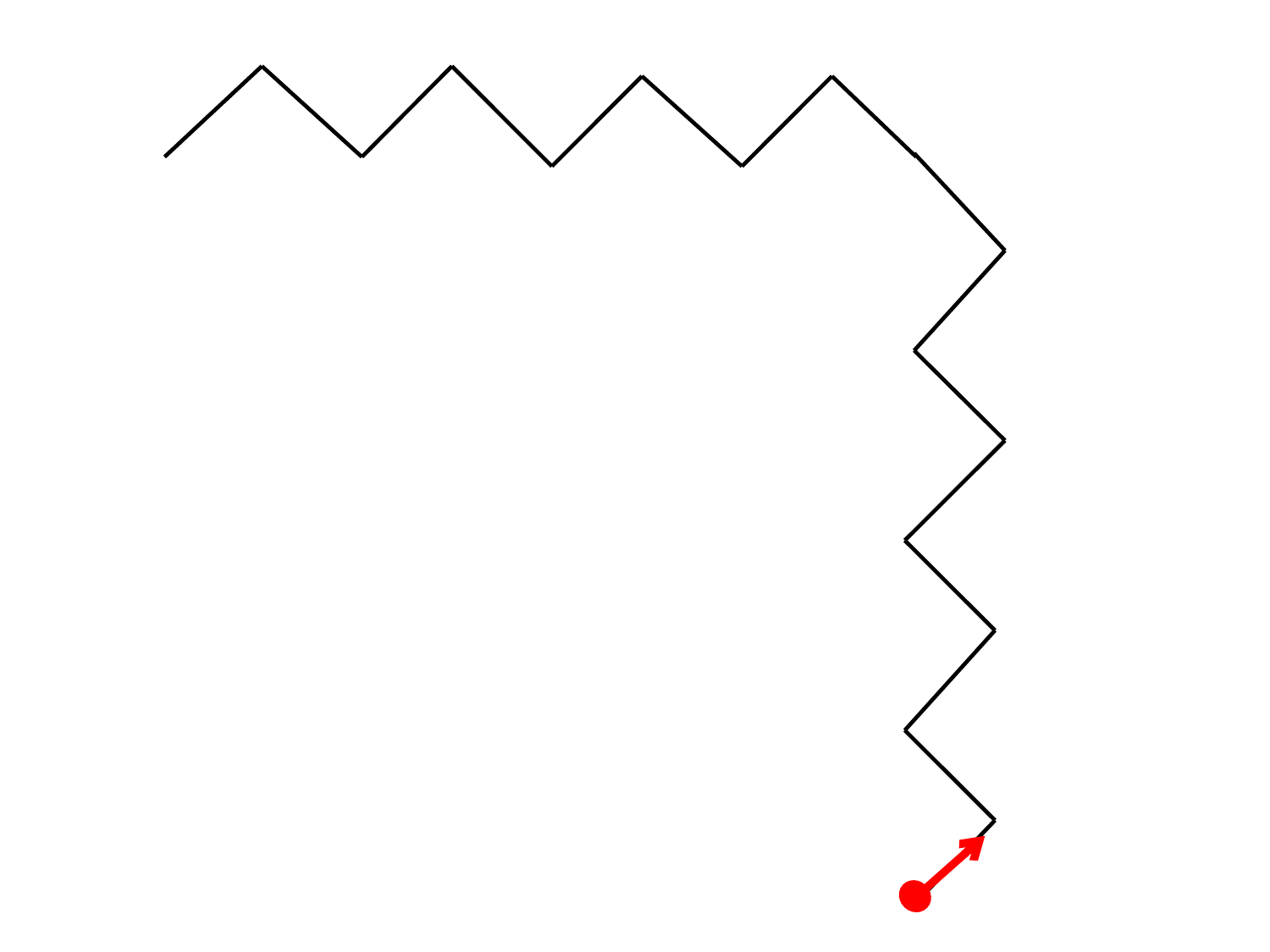

Supplement: Supplementary file 1 — Supplementary file1 (PPTX 69 KB) [file 221_2023_6627_MOESM1_ESM.pptx]
